# Research progress on the relationship between lung cancer drug-resistance and microRNAs

**DOI:** 10.7150/jca.31952

**Published:** 2019-11-17

**Authors:** Xuan Ma, Ai-Ling Liang, Yong-Jun Liu

**Affiliations:** Medical Molecular Diagnostics Key Laboratory of Guangdong & Departments of Biochemistry and Molecular Biology & Departments of Clinical Biochemistry, Guangdong Medical University, 523808, Dongguan, Guangdong, P.R. China

**Keywords:** microRNAs, lung cancer, drug-resistance, EGFR, p53, EMT, apoptosis

## Abstract

Lung cancer, a malignant tumor with the highest death rate of cancer, seriously endangers human health. And its pathogenesis and mechanism of drug resistance has been partially clarified, especially for the signal pathway of epidermal growth factor receptor (EGFR). The targeting therapy of EGFR signaling pathway in non-small cell lung cancer (NSCLC) has achieved a certain effect, but the two mutation of EGFR and other mechanisms of lung cancer resistance still greatly reduce the therapeutic effect of chemotherapy on it. MicroRNA is an endogenous non coding RNA, which has a regulatory function after transcriptional level. Recent studies on the mechanism of lung cancer resistance have found that a variety of microRNAs are related to the mechanism of lung cancer drug-resistance. They can regulate lung cancer resistance by participating in signal pathways, drug resistance genes and cell apoptosis, thus affecting the sensitivity of cancer cells to drugs. Therefore, microRNAs can be used as a specific target for the treatment of lung cancer and plays a vital role in the early diagnosis, prognosis and treatment of lung cancer. This article reviews the mechanisms of lung cancer resistance and its relationship with microRNAs.

## Introduction

In recent decades, lung cancer has become the most common malignant disease with the highest incidence and mortality rate in China and even the whole world, which poses a serious threat to human health. In 2016 Lachgar A *et al* 1 found that over 60% of lung cancer patients present with locally advanced or metastatic disease (stage III or IV) at the time of diagnosis, during this period that surgical resection may not be an option. Therefore, until recently, conventional chemotherapy and radiation therapy were the main types of treatments for lung cancer patients 2. However drug resistance in chemotherapy for lung cancer is complex and these mechanisms are not clarified yet, which seriously affects patients' clinical treatment effect and cause a very poor prognosis.

Over the past few decades, non-small cell lung cancer (NSCLC) and small cell lung cancer (SCLC) were the most common classification of diagnosing lung cancer.

This way doesn't require further morphological sub classification because of the limited treatment options. NSCLC comprises approximately 80%-85% of all lung cancers including adenocarcinoma and squamous cell carcinoma 2, 3. Thus NSCLC becomes the focus of studying lung cancer resistance mechanisms.

Nowadays there are several mechanisms of lung cancer drug-resistance mentioned in various literatures. In 2015 Wang *et al* found EGFR self-resistance mutation, T790M second mutation and the activation of the PI3K/AKT signal pathways 4. In 2007 Lim *et al* found that gene of phosphate and tension homology deleted on chromosome ten (PTEN) mutations and Axl overexpression 5. In 2005 Pao *et al* found that kirsten rat sarcoma viral oncogene (KRAS), v-raf murine asrcoma viral oncogene homolog B1 (BRAF) and human epidermal growth factor receptor-2 (HER-2) mutations 6. In 2007 Engelman found that mesenchymal-epithelial transition (MET) factor proto oncogene amplification and overexpression of protein 7. There are also some mechanisms concerned with signal pathways. For example, in 2014 Wu found that epithelial-mesenchymal transition (EMT) cell growth related to the deletion of signal pathways 8.

Most of the above-mentioned mechanisms of lung cancer resistance are related to gene mutation, deletion and amplification.

In addition to the above-mentioned lung cancer resistance mechanisms, there are other lung cancer resistance mechanisms involving pharmacokinetics and multidrug resistance genes, such as the following aspects. i) In 2016 Wei *et al* found that membrane transporter-mediated drug efflux pump mechanism, as the most common mechanism, which can reduce intracellular drug accumulation. This mechanism involves overexpressed membrane protein with efflux pump, such as ABC family member P-glycoprotein (P-gp), multidrug resistance-associated protein (MRP), lung resistance-related protein (LRP) and so on 9. ii) Intracellular abnormal enzyme system can be combined with a variety of chemotherapeutic drugs, which reduces drug activity, or enhances the DNA repair ability of tumor cells, and prevent the damage of chemotherapeutic drugs to the tumor cells 10. These intracellular abnormal enzymes are overexpressed topoisomerase, glutamyl transpeptidase, *O^6^*-methyguanine-DNA methyltransferase (MGMT) and so on. iii) In 2015 Javid *et al* found that anti-apoptotic effect of cells is enhanced, such as over-expression of anti-apoptotic genes bcl-2 and c-myc, which makes tumor cells less susceptible to apoptosis 11.

The epidermal growth factor receptor (EGFR) is a member of a family of four closely related receptors: EGFR (or erbB1), HER2/neu (erbB2), HER3 (erbB3) and HER4 (erbB4) 12. Approximately 50% of Asian patients with NSCLC have EGFR mutations 13. Patients with sensitizing mutations of EGFR, compared to conventional chemotherapy, these targeted therapy, such as epidermal growth factor receptor-tyrosine kinase inhibitors (EGFR-TKIs) have achieved huge success and became one of the standard treatment-regimes for NSCLC. However, patients ultimately develop resistance to these drugs, even though EGFR-TKIs have been established as the standard therapy for EGFR-sensitizing mutant advanced NSCLC clinically 14, 15.

According to the current mechanisms of lung cancer resistance, researchers have classified them into two categories: primary and acquired resistance. The primary resistance is defined as the failure to respond to the treatments at the first time after receiving EGFR-TKIs and presents no obvious improvement in symptoms, disease control or overall survival. Approximately 85% of all primary lung cancers are NSCLC. While some patients with activating EGFR mutations who are initially responsive to EGFR TKIs very well at firstly, eventually would be developed acquired resistance after a complete or partial response or ≥ 6 months of stable disease after treatment with a targeted therapy 16. Strategies to overcome these intrinsic and acquired resistance mechanisms are complex. Thus studies on the lung cancer drug resistance are extremely important in order to define the best treatment strategy.

MicroRNAs (miRNAs, miRs) are a class of small endogenous single strand non-coded RNA, about 19-25 nt, found in eukaryotes, and have the function of regulating after gene transcription 15. MicroRNAs' regulation mechanism is that the longer miRNAs primary transcript produces mature miRNAs through a series of nuclease shear processing. Then these mature miRNAs are assembled into the RNA-induced silencing complex (RISC). Then mature miRNAs recognize and combine the target genes or the target mRNAs' 3' terminal non translation region (UTR) by complementary pairing of bases. The RISC will conduct the process of mRNAs' degradation or inhibition of their translations. MiRNAs and target mRNAs combined incompletely can inhibit the expression of mRNAs at the level of protein translation. While when they may complement each other completely (or almost completely complementary), which can degrade the target mRNAs degradation and change the expression level of the target protein, then achieve the regulatory effect 17.

In 2016 Ren *et al* found that miRNAs can regulate a variety of target genes or one kind of miRNAs can be regulated by various genes 18. In particular, in 2006 George *et al* found that abnormal expression of miRNAs is probably one of the causes of tyrosine kinase inhibitors (TKIs) underlying mechanisms of primary resistance 19. In 2016 Thomas *et al* also found that several miRNAs can increase the therapeutic efficiency of EFGR-TKIs by regulating the drug sensitivity of cancer cells 20.

From the above literatures, it can be seen that miRNAs may play an important role in the treatment of lung cancer by regulating genes' expression, or by affecting the efficacy of drugs. Therefore, this article mainly reviews the role of miRNAs in several lung cancer resistance mechanisms based on recent literatures.

## Mutual regulation between overexpression of AXL and miRNAs in NSCLC

Axl (also known as AXL, UFO, ARK, or TYRO7) is one of the members of the receptor tyrosine kinase sub-family, and binds to the growth stagnation specific gene 6 (Gas6) to activate its tyrosine kinase activity. It mediates signal conduction from extracellular matrix to cells, and activates downstream signal transduction pathways, and participates in cell adhesion, proliferation, and apoptosis. The abnormal expression of Axl gene plays an important role in the tumorigenesis and development of many tumors 21.

In 2016 Chen found that miR-432 over-expression can increase the sensitivity of lung adenocarcinoma cells to cisplatin. E2F3 and AXL are targets for miR-432 acting on lung adenocarcinoma, and the target is 3' UTRs. It was found that miR-432 levels were negatively correlated with the levels of E2F3 and AXL. Increase miR-432 may reverse the resistance of lung adenocarcinoma cells to cisplatin by targeting to improve the therapeutic efficacy of lung adenocarcinoma patients 22.

In addition, in 2014 Wang *et al* found that overexpression of Axl significantly increased the expression level of miR-374a and decreased the expression of miR-548b in gefitinib-resistant NSCLC cells, Calu1 and HCC827-Gef cells. Then they found miR-374a induces EMT, colony formation and drug resistance in gefitinib-sensitive lung cancer cells. While silencing of miR-374a inhibits migration and invasion in gefitinib-resistant NSCLC. And miR-548b can target CCNB1 to control cell cycle progression in NSCLC cells 23. Following that we can infer that overexpression of Axl could increase drug resistance to gefitinib in NSCLC cells via mediating miRNAs involved with EMT or cell proliferation process.

In 2016 Cho *et al* had another interesting founding. They demonstrated that there may be a regulatory feedback loop between AXL and miR-34a at the post-transcriptional level. Both the GAS6-binding domain and the kinase domain of AXL are shown to be crucial for this autoregulation. They used bioinformatics and molecular techniques to reveal that miR-34a may target the 3' UTR of AXL mRNA to inhibit AXL express. Importantly, AXL overexpression may induce miR-34a expression by activating the transcription factor ELK1 via the JNK signaling pathway. That subsequently represses AXL expression to induce G1 arrest and cell apoptosis. Therefore, they assume not only that AXL could up-regulate miR-34a expression, but also that miR-34a could turn back to down-regulate AXL by directly targeting its 3' UTR 24.

It has been reported that AXL transcription can be induced by MAPK-AP1 activation and by MZF1 transcriptional activity in EGFR-TKIs 25. Axl may drive cancer cell growth through activation of several downstream pathways, such as MAPK, AKT and NF-kB, and Axl-mediated EGFR-TKI acquired resistance may occur during EMT 23. In 2012 Zhang *et al* also reported that AXL upregulation is the second most common mechanism of EGFR TKI acquired resistance (after EGFR T790M) in EGFR-mutant NSCLCs 26.

Based on the above literature, we can found that over-expressed Axl can not only regulate miRNAs or be regulated by miRNAs in an ex parte way, but also contrast a feedback loop with miRNAs to regulate each other's expression. This finding is interesting and significant because it offers another possibility in treating lung cancer patients. If the feedback loop really exists between miRNAs and targeted genes, we'd better avoid using this kind of miRNAs or targeted gene medicines singly, which may reduce the effect of the treatment in lung cancer. And this feedback loop explains the drug resistance mechanisms from another point of view, making it more completely. With the deepening of research on lung cancer, these findings provide a new way to understand the mechanisms of drug resistance in lung cancer cells.

## MiRNAs can mediate the deletion of PTEN to lead cell resistance

PTEN (or named mutated in multiple advanced cancers, MMAC1) is a tumor suppressor gene. It is the key regulator of cell proliferation, growth, differentiation and apoptosis 27. As early as 1994 Xu *et al* have found that the loss of PTEN is associated with the development and process of cancer 28. In 2006 Tang *et al* found that PTEN expression is down or absent in NSCLC 29.

PTEN enzymes are involved in transduction of signaling pathways. PTEN can transmit signals to cells, stop cell division and enter programmed cell death (or apoptosis). PTEN can catalyze PIP3 dephosphorylation. When PTEN is absent, PIP3 dephosphorylation is weakened, further activating PI3K/AKT pathway and inducing drug resistance. In 2010 Yamamoto found that the deletion of PTEN is due to the EGR1 shift of transcription factor PTEN, which is controlled by nuclear and further causes TKI resistant 30. In 2011 Derfoul *et al* found that miR-214 can regulate the tumor suppressor gene PTEN and regulate the proliferation and apoptosis of tumor cells. Inhibition of PTEN activates the PI3K/AKT pathway, thereby increasing the survival of tumor cells treated with drug chemotherapy 31.

MiR-92a has been proved to play an important role in many kinds of human cancers. In 2016 Ren *et al* found that miR-92a can promote cell proliferation, migration and invasion of NSCLC, and increase the drug resistance of NSCLC to chemotherapeutic drugs. Meanwhile the downregulation of miR-92a will have the opposite effect.

The researchers found that the miR-92a expression was higher in four different NSCLC cell lines (A549, SPCA1, H1299 and H358) than that in normal lung cells. By constructing the reported gene plasmid and transfecting it into the A549 cells, the researchers conducted Luciferase assay. They found that the expression of PTEN and miR-92a was negatively correlated. At the same time, Quantitative real- time reverse transcription PCR and Western blotting experiments also found that the levels of PTEN mRNA and protein in A549 cells were negatively correlated with the expression of miR-92a. And the relevant levels of PI3k and AKT were also negatively related to miR-92a. Therefore, it is inferred that PTEN is a direct target gene for miR-92a. The expression of miR-92a is increased when PTEN is deleted, meanwhile the PTEN of phosphorylation is reduced, and the PI3K/Akt signal pathway is activated to lead to drug resistance of lung cancer cells^18^.

In 2015 Xie *et al* transfected miR-106a mimics and knocked down miR-106a in three kinds of NSCLC cells to apply experiments. They found that miR-106a was up-regulated in NSCLC cell lines. Inhibition of miR-106a in NSCLC cells substantially inhibited cell proliferation, migration, and invasion. PTEN as the direct target of miR-106a, it was inversely correlated with PTEN in NSCLC cells. Their study demonstrated that miR-106a was significantly increased in NSCLC cell lines. Down-regulated expression of miR-106a could inhibit tumor growth and metastasis of NSCLC cells by increasing PTEN expression 32. A previous report showed that the PTEN/PI3K/pAkt pathway may play an important role in lung cancer 33.

Taken together, these findings indicated that miRNAs could promote growth, metastasis, and chemoresistance in NSCLC cells at least partially by targeting PTEN. Until now, drug resistance mechanisms in lung cells referred to the deletion of PTEN, at least PI3K/Akt signaling pathway is of great significance in this part. While as for the drug resistance in lung cancer, there must be more profound and complicated causes involved in the mechanisms need to be explored in the future.

## MiRNAs mediate cell resistance in lung cancer via apoptosis

Apoptosis is a gene-encoded programmed cell death. Under various physiological conditions, it is regulated by intracellular and/or extracellular signals and characterized by morphological changes of the cells targeted for death 34.

Caspase family includes apoptotic initiators in the upstream of the cascade reaction, such as Caspase-2, Caspase-8, Caspase-9, and Caspase-10 and so on. These factors can self-activate and then identify and activate downstream caspase members with the help of other proteins. For example, Caspase-8 can activate Caspase members in the downstream of almost all apoptosis cascade reactions and induce apoptosis, which lead to cascade amplification effect of apoptosis 35, 36.

Bcl-2 and NF-κB are recognized as anti-apoptosis genes. The Bcl-2 family contains at least 17 or more members. A large number of studies have confirmed that there are Bcl-2 and NF-κB super-expressions in lung cancer, gastric cancer, ovarian cancer, and colon cancer. Bcl-2 protein is a product of Bcl-2 pro-oncogene encoding, which promotes the survival of cancer cells and inhibits apoptosis. Their anti-apoptosis effects play an important role in the malignant proliferation of tumor cells and are closely related to invasion and metastasis 37, 38.

According to the structure and function, the Bcl-2 family can be divided into three groups. The first group is anti-apoptosis or apoptosis family, including Bcl-2, Bcl-XL, Mcl-1, Bcl-w and A1. They all contain 4 BH domains (BH1-4). The second group is multiple BH domain apoptosis family, including Bax and Bak, which contains multiple BH domains. The third group is only BH3 domains families which promote apoptosis. They contain only BH3 domains that are homologous to Bcl-2, including Bad, Bid, Bik, Bim, Bmf, Hrk, Noxa, PUMA, and Beclin-1 39. Bax and Bak's BH3 domains can form heterodimers with Bcl-2 and Bcl-xl, promoting apoptosis 40. At the protein level, different Bcl-2 family proteins can interact to form different dimers, thus antagonizing or enhancing the function of apoptosis 41. Therefore, some researchers proposed that the ratio between inhibition factors and activation factors may determine the tendency of cells to apoptosis 42, 43. When apoptosis is suppressed, it may lead to malignant lesions and drug resistance in cancers. Under poor conditions (such as DNA damage or hypoxic oxygen), tumor suppressor genes such as P53 can trigger apoptosis 44 .

P53 is one of the most common mutation genes in human tumors. P53 gene mutations and functional defects are about 45%-70% in NSCLC. It is an important cause of tumor occurrence and treatment failure 45. Wild p53 function may be regulated by p53 apoptosis stimulating protein. The latter protein combines with P53 to form a complex to act on the original apoptosis gene. Then that promotes p53 dependent apoptosis by increasing the activity of the original apoptosis gene 46.

The process of apoptosis is complex and involves a variety of protein families, aforementioned caspase family proteins, the Bcl-2 family proteins, and the p53 protein, which can be divided into two categories according to their functions in apoptosis — anti-apoptosis and pro-apoptosis. We can find that these proteins could be mediated by miRNAs to perform the two different functions to regulate the process of drug resistance in lung cancer.

Caspase-3 is one of the most important executors of the caspase family. It is the main effect factor in the apoptosis process. Its activation is a sign that apoptosis has entered an irreversible stage 47.There is a study found that the activity of cysteine caspase-3 in NSCLC cells after transfection of miR-92a mimics was significantly reduced. And the activity of caspate-3 was significantly enhanced after transfection of anti-miR-92a. It is shown that miR-92a can promote cell proliferation and reduce apoptosis in NSCLC. It is speculated that overexpressed miR-92a can increased the resistance of NSCLC cells through down-regulated caspase family related genes 18.

In 2015 Cui *et al* demonstrated that miR-244 markedly enhanced proliferative and migratory effect of NSCLC cells. They found the expression of miR-224 negatively correlates with the expression of Caspase-7 and Caspase-3 in tissue samples from patients with lung cancer, which also induced drug resistance in NSCLC. However miR-224 regulates Caspase-3 at translation levels and Caspase-7 in lung cancer remains largely unexplored. At least miR-224 promotes lung cancer cell growth and migration partially by inhibiting Caspase-7. Then they also confirmed that up-regulated miR-224 expression in NSCLC might be partially controlled by NF-κB signaling through binding of RELA/p65 to miR-224 promoter region. Hence, they suggest that NF-κB/miR-224/CASP-3, -7 pathway might be an ideal target for therapeutic intervention in certain lung cancer patients 48.

In 2016 Wu *et al* found that miR-301b was highly expressed in lung cancer tissues and cell lines. Expression of miR-301b was induced by hypoxia, and miR-301b suppressed expression of Bim by targeting its 3' UTR. Their results demonstrated that hypoxia-induced microRNA-301b expression enhances cell growth and reduces apoptosis by regulating Bim expression 49.

Erk1/2 pathway controls several cellular functions, such as proliferation, survival and migration, these all are closely related to drug resistance in cancer 50. In 2012 Romano *et al* showed a link between the Erk1/2 pathway and Bim expression through miR-494 in NSCLC. MiR-494 was the most down-regulated miRNA after Erk1/2 inactivation. They assumed that axis PED-ERK-miR-494-BIM existed in NSCLC cells. And then they found that the overexpression of this microRNA in H460 tumor necrosis factor (TNF)-related apoptosis-inducing ligand (TRAIL)-sensitive cells, by down-regulating BIM, increased the resistance to TRAIL induced apoptosis. The same result was obtained on TRAIL-resistant A549 cells, the down-regulation of miR-494 made A549 more sensitive to TRAIL-induced apoptosis, confirming the relevant role of miR-494 in TRAIL resistance. That means miR-494 could induce TRAIL resistance through the down-modulation of Bim to increase resistance in NSCLC. 51.

In 2018 Choi *et al* found expression of P53, and Notch2 were decreased in miR-93-5p over-expressed lung cells. And expression of Bcl-w or P21 was also negatively correlated with that of miR-93-5p in lung cancer tissue samples 52. The tumor suppressor p53 binds the promoting survival Bcl-2 family proteins such as Bcl-w 53. We can infer that there is a Bcl-w-P53/Notch2-P21 signaling axis to mediate cells growth and premature senescence in lung cells. These cell behaviors may furthermore increase drug resistance in lung cancer.

In 2018 Baumgartner *et al* explored 17 miRNAs are dysregulated following PI3K/AKT inhibition of EGFR mutant NSCLC cells. They used bioinformatics to analyze then they found that dysregulated miRNAs act in a concerted manner to enhance the activity of the EGFR signaling pathway, such as PI3K/AKT, KRAS/ERK and JAK/STAT signal pathways. These findings were closely mirrored by attenuation miR-19b in NSCLC cell lines which resulted in reduced phosphorylation of ERK, AKT and STAT and effector proteins in EGFR mutant NSCLC cells.

They found that serine/threonine phosphatase PP2A subunit PPP2R5E and BCL2L11 encoding Bim were identified as major targets of miR-19b by target validation assays. MiR-19b potentiates EGFR signaling by targeting PP2A subunit PPP2R5E and confers apoptosis resistance by targeting BCL2L11 encoding the BH3 domain containing protein Bim. MiR-19b induces proliferation and apoptosis resistance by targeting PPP2R5E and BCL2L11. It can be inferred that molecular processes of EGFR signaling involving miRNAs may provide insights into improving the management of EGFR-mutant lung cancer patients treated with TKIs 54. The proapoptotic BH3-only protein Bim is a master regulator of cell death in cancer cells 55, which is a relevant target of miR-19b in spontaneous and decrease drug resistance in lung cancer.

From the above literature, we can see that miRNAs can regulate the relevant apoptosis proteins or genes at different nodes in signaling pathways through their own over-expression or downward expression. These apoptosis family members, such as Bim, P53 and Caspase3 and the like, have already been a research hot topic for a long time, and some mechanisms are pretty clear now. The decrease of apoptotic ability is accompanied by the increase of drug resistance in lung cancer cells. The drug resistance of lung cancer cells is regulated by enhancing the ability of cells to anti-apoptosis or pro-apoptosis. The role of cell apoptosis in drug resistance of lung cancer has not been elucidated until now, however these documents are sufficient to indicate a relationship between both. As the research further develops, we can use apoptosis to regulate miRNAs to make lung cancer cells resensitive to drugs, improving efficacy of medicine in lung cancer patients.

## MiRNA-200 family mediate drug resistance by EMT in lung cancer

EMT is a fundamental cellular process that plays critical roles in development, cancer metastasis and tissue wound healing 56. In the process of drug resistance, the morphological changes of epithelial cells to mesophyll cells are often accompanied by the decrease of epithelial markers and the increase of mesophyll markers 57. At the same time, it is often accompanied by Axl activation, transformation growth factor-β (TGF-β) activation 58. ZEB1 (zinc finger E-box binding homeobox 1) is the key molecule for EMT conversion, an important activator in EMT inhibiting expression of basement membrane components and cell polarity factors 59. And EMT is characterized by a switch from an epithelial phenotype of polarized cells with expression of epithelial markers such as E-cadherin to a mesenchymal phenotype of cells that lack polarity, are motile and have down regulation of E-cadherin expression accompanied by an increase in Vimentin 60, 61.

As early as 1994, Nichterlein *et al* found that miR-452 can exert anti-tumor activity by inhibiting the EMT of NSCLC cells 62. In 2011 Roybal *et al* found that miR-200 can inhibit the invasion and expression of lung adenocarcinoma cells by inhibiting the expression of target gene Flt1/VEGFR1 63. The EMT process and sensitivity to EGFR-TKIs therapy of bladder cancer cells can be regulated by regulating the expression of target gene ERRFI-1 64. In another study, researchers screened miRNAs associated with kzoltinil resistance H1322 cells in NSCLC found that the reduction of miR-200c was likely related to EMT 65. About 20%-44% of treated NSCLC patients acquire resistance to EGFR-TKIs through phenotypic changes of the tumor cells undergoing EMT, possibly as a result of altered epigenetic regulation 66. It has been shown that EMT-related acquired resistance to EGFR-TKIs in NSCLC is driven by increased ZEB1, which is negatively regulated by miR-200c 67, 68.

In a recent study, miR-200c and Cathepsin L were proved to reciprocally influence each other, regulating paclitaxel resistance through EMT in A549 cells 69. In 2013 Ahmad A *et al* found that pretreatment with miR-200b and let-7c in TGF-β1-induced A549 cells, about 67.69% inhibition of TGF-β1-mediated effect on erlotinib resistance was observed. Re-expressing of miR-200b and let-7c could also downregulate ZEB1 and remarkably break-off the inhibition of E-cadherin expression, both of which were the index to the reversal of EMT 60. Except erlotinib, gefitinib is another EGFR-TKI that is often used in TKI therapies to treat NSCLC patients. And in 2015 Zhen Q *et al* found that the expression of miR-200a was observed to render NSCLC cells much more sensitive to gefitinib treatment 70.

MiR-200 is a family of tumor suppressor miRNAs consisting of five members, which are significantly involved in inhibition of EMT, repression of cancer stem cells (CSCs) self-renewal and differentiation, modulation of cell division and apoptosis, and reversal of chemoresistance 71. Phenotypic changing is one of the characters of cancer cells, such as EMT. It is because of the appearance of this kind of biological behavior, which makes cancer cells get the greater ability of migration and invasion. So the cancer cells are more likely to develop resistance. From the above literates we can found the countless ties between the EMT and the miR-200 family, it can be inferred that EMT plays an important role in lung cancer resistance, but the specific mechanism has not been clarified. Besides miR-200 family, it is also not known whether EMT is related to other genes or miRNAs. But it's more important to focus on research about miR-200 family; this may reveal the root cause of EMT, to solve the problem of cancer cell migration and invasion at the source. If it can be targeted to inhibit EMT through miRNAs, it may be possible to find new treatments for resistance to lung cancer, making the most of medicine treatment.

## MiRNAs and other resistance mechanisms in lung cancer

### The mechanism of universal lung cancer resistance and miRNAs

As mentioned earlier, the universal drug- resistance mechanism for lung cancer involves drug metabolic kinetics, which includes MRP, LRP and Breast cancer resistant protein (BCRP). These four membrane transporters cause drug resistance by reducing intracellular drug concentration or changing the intracellular distribution of drugs 72.

LRP/MVP is a ribonucleoprotein particle able to transport drugs such as platinum derivatives from the nucleus to the cytoplasm. After that, these compounds can be transported outside from the cells by ABC-transporters 73-75. In 2016 Janikova *et al* immunohistochemically evaluated expression of P-gp, MRP and LRP/MVP and quantified the relative levels of miR-23b in 62 NSCLC patients' samples. Their results showed that miR-23b is mostly downregulated in NSCLC samples (57/62). Their findings indicate that the risk of death or relapse in NSCLC patients with downregulated miR-23b increases together with LRP/MVP expression and decreases in patients with upregulated miR-23b 76. Expression of P-gp, MRP and LRP/MVP are often observed in NSCLC samples and it is believed that these molecules play a major role in the emergence of multidrug resistance 77-79.

### APC methylation or involvement in lung cancer resistance

The APC (adenomatous polyposis coli) gene can control the death or growth of cells. This gene encodes a tumor suppressor protein that acts as an antagonist of the Wnt signaling pathway. It is also involved in other processes including cell migration and adhesion, transcriptional activation, and apoptosis 80. Abnormal methylation of APC gene promoter occurs in lung cancer patients. In 2016 Feng *et al* found that APC gene promoter methylation is associated with the clinical characteristics of tumor patients, mainly tumor staging, lymph node metastasis and smoking 81. While in NSCLC β-catenin and APC mutations are uncommon 82.

Resistance of the same cell line to paclitaxel was significantly associated with miR-135a expression. In both *in vitro* and *in vivo* models, researchers observed that inhibition of miR-135a expression led to re-sensitization of previously resistant NSCLC cells to paclitaxel and caused the cells to undergo apoptosis. Expression of miR-135a has also been linked to the activity of the APC gene, which is involved in cancer development 83.

### Resistance to lung cancer related to TRAIL

TRAIL, a cytokine and a member of the TNF family, is being tested in clinical trials as a powerful anticancer agent. Although TRAIL had shown clinical efficacy in a subset of NSCLC patients, acquired resistance to this anticancer agent undermines its therapeutic value. The mechanism of this resistance is still not fully understood 84.

In 2008, Garofalo *et al* reported that NSCLC cells overexpressed miR-221/222 were TRAIL-resistant and showed an increase in migration and invasion capabilities 85. Later on, the same group demonstrated that miR-34a and miR-34c, which are downregulated in NSCLC cell lines, could play a significant role in lung carcinogenesis by modulating the expression of PDGFR-α/β(platelet derived growth factor receptor-α/β) and thereby regulating TRAIL-induced cell death sensitivity 86. Expression of miR-21, miR-30c and miR-100 in NSCLC has been related to acquire TRAIL resistance 87. Accordingly, continuous exposure to TRAIL caused acquired resistance to this agent in the future.

### Low expression of ATM could increase sensitivity to drugs in lung cancer

The ataxia telangiectasia-mutated gene (ATM) is an important gene related to DNA damage testing. The ATM protein and the closely related kinase ATR (ataxia telangiectasia and Rad3 related, ATR) are thought to be master controllers of cell cycle checkpoint signaling pathways that are required for cell response to DNA damage and for genome stability. It directly perceives DNA double-strand fracture damage and initiates many DNA damage signal response pathways. P53 can be activated by phosphorylation and then transcribed to activate the expression of cell cycle checkpoint protein P21 to participate in the process of regulating DNA damage and repair 88. In 2017 Allison Stewart *et al* found that schlafen family member 11 (SLFN11), EMT, and ATM mediate therapeutic response in SCLC to novel targeted agents such as PARP inhibitors. They found that knockdown of SLFN11 and ATM directly alters drug sensitivity. Silencing of ATM increased the sensitivity of DMS79 and H209 cells to cisplatin, talazoparib and olaparib in SCLC 89.

The drug resistance mechanism of lung cancer is complex and involves many proteins, genes, and miRNAs. A couple of examples above are all novel drug resistance mechanisms related to lung cancer. For instance, PDGFR-α/β is a kind of platelet-derived growth factor. This gene is essential for normal development of the cardiovascular system and aids in rearrangement of the actin cytoskeleton. Another one, APC gene is involved in Wnt signal pathway, which is related to the origin of cancer in humans. All of the above provide new perspectives for the study of drug resistance of lung cancer. It is believed that with the deepening and expansion of research, the roles of more and more genes, proteins and miRNAs can be elucidated in the future, to provide effective evidence for future drug resistance of lung cancer.

## Summary

In the lung cancer, miRNAs perform dual regulatory function: they act as oncogenes to promote cancer development or as tumor suppressors to inhibit this process 84. As stated above miRNAs overexpressed in lung cells (or drug-resistance lung cells), such as miR-374a, miR-92a, miR-106a, act as oncogenes that promote the development of lung cancer cells and increase drug resistance by negatively regulating tumor suppressor genes and/or genes that control cellular processes such as EMT and apoptosis. Other miRNAs do the opposite function when they are overexpressed in the lung cells, such as miR-548b, miR-200a, and miR-432. And there are some miRNAs such as miR-34a can form a negative feedback closed- loop with the expression of the related gene Axl. And miR-34a and Axl are reciprocally linked in a feedback loop to regulate each other in the lung cancer cells, which can affect drug resistance of lung cancer cells.

The various miRNAs expression patterns are unique for specific resistance mechanisms in lung cancer. These molecules are either over- or under expressed depending on the resistance mechanism types^90^, ^91^. Table 1 summarizes the effects of miRNAs in resistance mechanisms of lung cancer in this review.

The relationship and mechanism of miRNAs with lung cancer resistance is complex. And many drug resistance mechanisms have not been elucidated. A lot of experimental evidence suggests that single mechanism is not fully enough to explain the emergence of lung cancer resistance. According to the above literature, we can find that these lung cancer drug resistances are often concerned with many other mechanisms, such as pharmacokinetics, EMT, cell cycle and cell apoptosis and so on (Table 2). These factors interact with each other, leading to drug resistance in lung cancer.

MiRNAs have been involved in a variety of signaling pathways, such as JNK/ELK1 signal pathway, PI3K/Akt signal pathway and NF-κB signaling pathway and so on. The capacity of a single miRNA to regulate the expression of multiple genes simultaneously presents an opportunity to use these small molecules in personalized therapy as individualized therapeutic tools. MiRNAs may not only be used as markers of the response to cisplatin but also as potential treatment tools that stimulate the sensitivity of lung cancer cells to this chemotherapeutic agent 84. And they also have a very important guiding significance in avoiding and reversing lung cancer resistance effectively in the future. It is hoped that the miRNAs based targeting therapy can be applied in the future with more and more in-depth study of lung cancer cells and their drug resistance mechanism. Then we can effectively slow down the drug resistance of lung cancer patients and bring new opportunities for the treatment of patients, to improve a disappointing overall survival in lung cancer.

## Figures and Tables

**Table 1:** Effects of miRNAs in resistance mechanisms of lung cancer

miRNA	miRNA expression quantity	Downstream factors(target genes)	Regulatory relationship with downstream factors	Mechanism	Response to drugs	cell	References
miR-432	over-expression	E2F3 and AXL	inverse correlation	arresting cell cycle into S phase	increased sensitivity to cisplatin	lung adenocarcinoma cells	22
miR-374a	over-expression because of AXL	AXL (over- expressed)	positive correlation	induce EMT, migration , invation, colony formation	increased resistance to gefitinib	gefitinib-resistant NSCLC cells, Calu1 and HCC827-Gef cells	23
miR-548b	down -expression because of AXL	AXL (over- expressed)	inverse correlation	induce EMT, migration , invation, colony formation	increased resistance to gefitinib	gefitinib-resistant NSCLC cells, Calu1 and HCC827-Gef cells	23
miR-548b	over-expression	CCNB1	inverse correlation	cell cycle	increased sensitivity to gefitinib	NSCLC cells	23
miR-34a	over-expression because of AXL	AXL	mutual negative adjustment	JNK/ELK1 signaling pathway, cell cycle and cell apoptosis	increased sensitivity to drugs	lung adenocarcinoma cells (CLI cell lines)	24
miR-92a	over-expression	PTEN	inverse correlation	PI3K/Akt signal pathway	increased resistance to drugs	four different NSCLC cell lines (A549, SPCA1, H1299 and H358)	18
miR-92a	over-expression	caspase-3	inverse correlation	cell apoptosis	increased resistance to drugs	NSCLC cells	18
miR-106a	over-expression	PTEN	inverse correlation	PI3K/Akt signal pathway	increased resistance to drugs	three kinds of NSCLC cells	32
miR-244	over-expression	Caspase-7 and caspase-3	inverse correlation	NF-κB signal pathway	increased resistance to drugs	NSCLC cells	48
miR-494	over-expression	Bim	inverse correlation	MEK/ERK or Erk1/2 pathway(PED-ERK-miR-494-BIM)	increased resistance to TRAIL	NSCLC cells	51
miR-93-5p	over-expression	Bcl-w, P21, P53	inverse correlation	cell apoptosis	increased resistance to drugs	lung cancer tissue samples	52, 53
miR-19b	over-expression	PPP2R5E and BCL2L11	inverse correlation	EGFR mutation and cell apoptosis	increased resistance to TKIs	NSCLC cells	55
miR-200c	Low-expresstion	ZEB1	inverse correlation	EMT	increased resistance to EGFR-TKIs	NSCLC cells	64, 66, 67
miR-200c	over-expression	Cathepsin L(CTSL)	mutual negative adjustment	EMT	increased sensitivity to paclitaxel	A549 cells	68
miR-200b and let-7c	over-expression	ZEB1and E-cadherin	inverse correlation	EMT	increased resistance to erlotinib	NSCLC cells	69
miR-23b	low-expresstion	LRP/MVP	inverse correlation	drug metabolic kinetics	increased resistance to drugs	NSCLC samples	76-79
miR-135a	low lexpresstion	APC gene	inverse correlation	cell apoptosis	increased sensitivity to paclitaxel	NSCLC cells	83
miR-34a and miR-34c	over-expression	PDGFR-α/β	inverse correlation	cell apoptosis	increased sensitivity to TRAIL	NSCLC cell lines	86

**Table 2:** Drug resistance mechanisms in lung cancer

Drug resistance mechanisms	Cause of drug resistance	References
EGFR self-resistance mutation	Gene mutation	4
T790M second mutation	Gene mutation	4
activation of the PI3K/AKT signal pathways	Intervene signal pathways	4
gene of phosphate and tension homology deleted on chromosome ten (PTEN) mutations	Gene deletion (gene mutation)	5
Axl overexpression	Abnormal gene expression	5
KRAS, BRAF, HER-2 mutations	Gene mutation	6
MET factor proto oncogene amplification and overexpression of protein	Abnormal gene expression	7
EMT	Deletion of signal pathways	8
membrane transporter-mediated drug efflux pump mechanism, such as P-gp, MRP, LRP	Overexpressed membrane protein with efflux pump, related to pharmacokinetics	9
Intracellular abnormal enzyme system	Reduce drug activity and enhances the DNA repair ability	10
genes bcl-2 and c-myc over-expression	Cell apoptosis	11
